# What about Health Education? Hegemony, paradigms in tension and alternatives

**DOI:** 10.3389/fmed.2023.1289865

**Published:** 2023-11-09

**Authors:** Matías Blaustein, Fernando Miguel Garelli

**Affiliations:** ^1^Instituto de Biociencias, Biotecnología y Biología Traslacional (iB3), Departamento de Fisiología, Biología Molecular y Celular (DFBMC), Facultad de Ciencias Exactas y Naturales (FCEyN), Universidad de Buenos Aires (UBA), Buenos Aires, Argentina; ^2^Consejo Nacional de Investigaciones Científicas y Técnicas (CONICET), Buenos Aires, Argentina; ^3^Grupo de Didáctica de las Ciencias (IFLYSIB-CONICET), La Plata, Argentina; ^4^Departamento de Educación, Universidad Nacional de Luján, Luján, Argentina

**Keywords:** health education, hegemonic medical model, vertical paradigm, democratic paradigm, popular education

## Introduction

Health Education (HE) is a field that, despite being widely—almost intuitively—regarded as crucial, is not usually addressed in other health-related fields and health research agendas, leaving its role and implications relegated (1).

Both health communication and community participation in health share a similar taste, ubiquitously considered important but insistent and persistent as problematic. As Morgan once described it as a perpetual allure and a persistent challenge (2).

In this article, an overview of the HE field is shared, providing a brief sample of research and key ontological and epistemological stances in order to describe HE paradigms and perspectives in tension. This typology of perspectives may help to question and analyze which HE is being—implicitly or explicitly—supported by different health initiatives. Some experiences and theories from Latin America are also shared, which may not be very well known in other geographies, and these frameworks are placed in dialogue with others fostered in the Global North. All of this we hope may contribute to discussing questions such as how can health education (HE) contribute to broader health initiatives? How is HE performed in different educational contexts? Which HE do we have and which do we want?

## Health education research worldwide and the hegemonic medical model

A general overview of recent HE research, across countries, decades, and theoretical and analytical perspectives, can show a critique of what may be referred to as a biomedical perspective. Roughly, considering diverse references, a biomedical HE approach can be defined as one that solely considers biology and medicine excluding epistemological, anthropological, historical, social, and cultural frameworks, among others (3).

In South America, Martins et al. (3) analyzed a corpus of 169 scientific manuscripts from around the globe, finding a biomedical approach as the most disseminated. In Argentina, Revel Chion et al. (4) have shown that health is usually approached in high schools from a simplified and solely biological perspective. In a more quantitative and extensive study, including over 6,000 teachers from 16 countries in Europe, Africa, and the Middle East, Carvalho et al. (5) concluded that health promotion instead of the biomedical model should be considered, specifically in teacher training curricula. In consonance, Gavidia Catalán (6) has advocated for changes in HE in Spain, criticizing the traditional perspective centered upon a hygienist, biomedical view and pointing toward the creation of health-promoting schools. In Italy, Civitelly et al. (7) argued for a global health movement that should transcend solely biomedicine and become transdisciplinary and multi-method.

This sample of the literature illustrates a widespread critique of biomedical HE by researchers which poses two questions: Why is biomedical HE the dominant perspective? What other forms of HE can be considered? Understanding the reasons underlying the dominance of biomedical HE is a task that may be answered in a seemingly simple way but with complex, profound, implications: it is so because it is part of the naturalized dominant model in health worldwide. In order to develop this idea, the studies of Eduardo Menéndez, an anthropologist well known in Latin American Academia though mostly ignored in other latitudes and English-written literature, are considered.

This author has proposed, described, and analyzed what he has called the hegemonic medical model (HMM) over the past 50 years. The HMM can be promptly described as a group of practices, knowledge, and theories generated by the development of what is known as scientific medicine (8). This model can be traced to the end of the eighteenth century in Occident, and since then, it has successfully established other forms of knowledge and practices in health as subaltern (e.g., dominated, marginalized, and devalued), accomplishing a full identification with the only effective way to treat disease.

The main characteristics of the HMM are biologism, individualism, ahistoricity, asociability, positivism, mercantilism, pragmatic efficacy, asymmetry, authoritarianism, passive and subordinated participation of people (patients), juridical legitimacy, and identification with scientific rationality (8). According to Menéndez (8), biologism, i.e., the biomedical perspective, is its main structural characteristic, one which warrants not only the scientificity of the model but also its differentiation and hierarchy with respect to other perspectives. In the context of this article, mercantilism should be underlined as a second main feature because it refers to the intricate relationship between the HMM and private commercial interests. This link relates biomedicine with a general disempowerment of the population which delegates health to the medical systems and transnational corporations, responsible for the production of most medical drugs. This, in turn, affects the research agenda, largely shaped by these big pharmaceutical corporations, focusing on certain diseases and research topics (9, 10).

This strong identification between biomedicine and the hegemonic, naturalized, dominant model in health explains its rooting in HE (and other disciplines as well). Of course, other models exist and interact in conflict with the HMM. Menéndez recognizes two other models in tension with the HMM, the alternative medical model and a model based on self-support (8). The treatment of these exceeds this manuscript though their recognition underlines the existence of different perspectives in contradiction.

## Health education paradigms

Different conflicting perspectives in HE are focused in the study. In order to do this, Breilh's framework is considered for analyzing the field of epidemiology (11). Breilh shares a Bourdieuian perspective, conceptualizing health as a social field with different paradigms in conflict, each with its own definitions, methods, and practices. In this view, HE may be considered as a field in which a struggle between different ways of enunciating and acting occurs, which is in direct relation to social interests in conflict. Following Breilh (11), we may analyze these conflicting perspectives in HE according to three interdependent dimensions: ontological (what/how is Health?), epistemological (which are the valid ways of knowledge in health? how is knowledge built?), and praxic (what pedagogic/didactic stances do we consider in HE?). We will describe two different conflicting views in HE, the vertical and democratic paradigms, each with two different perspectives (12). This typology is an analytical tool that should be understood as such: the specific practices of individuals, such as teachers or doctors, may include combinations of these approaches and even vary in different contexts or situations.

The vertical HE paradigm is the dominant, biomedical view, linked to the HMM. Historically its main perspective has been hygienism (13), especially in the first half of the twentieth century, a view in which health is considered as the absence of disease; biomedicine is the only form of knowledge, and a monological transmission-reception pedagogical model is enforced (14). Within this paradigm, over the past 50 years, a more behavioral perspective has been fostered, which includes a wider conceptualization of health as bio-psycho-social equilibrium but is epistemologically and praxically equivalent to the hygienist perspective. In this view, healthy lifestyles, including exercise, nutrition, and interpersonal relations, are the main focus though it continues to be mainly normative, vertical, and decontextualized (15).

Subordinated and antagonistic to the verticalist paradigm, a democratic paradigm exists in the HE field. Despite being favored in academic HE circles, it is socially far less developed. Extending the considerations of Martins et al. (3), Jensen (13), and Fainsod and Busca (14), two different perspectives are distinguished in the study. On the one hand, the socioecological approach shares a more multidimensional view of health and an interdisciplinary epistemological stance. From this viewpoint, education is usually framed in a constructivist perspective (16), in which knowledge is not to be imposed but constructed. On the other hand, a critical-participative perspective may also be distinguished, where health is understood as a complex and polysemic object with diverse forms of knowledge considered as valid, seeking a dialogue between science, popular, and ancestral knowledge forms. In the praxical dimension, critical pedagogies such as popular education (17) tend to be favored.

## Discussion

So, what about health education? As concluding remarks, some questions are proposed, hoping more strongly to open a debate than to share answers. Which HE do we want? Which should we endorse? Can HE be more thoroughly included in health research agendas? What should be its role? These questions and their potential answers are not neutral. Even not answering or not addressing them is not neutral, their invisibilization only reinforces the dominant paradigm.

Furthermore, what type of HE do we have and which do we want in current healthcare challenges, such as mental health, eating disorders, environmental health, problematic consumption, or vector-borne diseases? What role did we attribute to HE in the COVID-19 pandemic? In a prior study, we argued that the main perspective enforced during the pandemic was hygienist HE (12). Could other approaches have helped diminish morbidity and mortality?

The democratic paradigm can offer answers to these important questions based on its more integral and participative approach to health. As it is rarer, a few theoretical frameworks are discussed that may add to its comprehension, specifically taking the critical-participative perspective into focus. This paradigm can be linked with a number of scientific education frameworks such as science education for social justice [e.g., (18)], activist science and technology education (19), and critical health literacy (20). These approaches coincide in that they are based on a critical view of reality, not only providing mainstream knowledge and practices but also opportunities to question, challenge, and reconstruct knowledge with the intention to transform both learners and their context.

The critical-participative perspective is pedagogically founded in popular education, an educational tradition based on the studies of Paulo Freire, which proposes a political, critical, dialogical, and transformative pedagogical framework (17). HE experiences based on this tradition contribute to considering health from a multidimensional human rights-oriented perspective, seeking community participation and contributing to its autonomy (21). This view of health can also be expanded considering critical epidemiology, a part of the collective health movement, which, very briefly, posits social determination of health as its main ontological stance, promoting dialectical, complex, and critical thinking with its potential for an emancipatory praxis (11).

These HE perspectives aim to empower the population, and, therefore, they entail the possibility to significantly improve health initiatives at a low cost, displaying non-commercial solutions and actions and seeking to improve individual and collective health. Can these intentions be put into a much wider, general practice? The allure is undeniable, but the challenge persists.

## Author contributions

MB: Writing – review & editing, Supervision, Validation, Resources, Funding acquisition. FG: Conceptualization, Investigation, Writing – original draft, Writing – review & editing.
